# Editorial: Evolving strategies in radiation therapy for benign intracranial tumors: current techniques, clinical challenges, and future prospects

**DOI:** 10.3389/fneur.2025.1691115

**Published:** 2025-09-24

**Authors:** Zhenyu Xiong, Kaida Yang, Tsuicheng Chiu

**Affiliations:** ^1^Department of Radiation Oncology, Rutgers Cancer Institute, Rutgers, The State University of New Jersey, New Brunswick, NJ, United States; ^2^Department of Radiation Oncology, Mount Sinai Hospital, New York, NY, United States; ^3^Department of Radiation Oncology, University of Texas Southwestern Medical Center, Dallas, TX, United States

**Keywords:** radiation oncology, radiotherapy, benign intracranial tumors, meningioma, treatment outcome

Benign intracranial tumors, such as meningiomas, vestibular schwannomas, pituitary adenomas, craniopharyngiomas, hemangioblastomas, and glomus tumors, represent a distinctive challenge in modern neuro-oncology. Although histologically non-malignant, these tumors are often situated in proximity to critical neural and vascular structures and can cause significant morbidity through visual loss, hearing impairment, neurological deficits, or endocrine dysfunction. Surgical resection remains the gold standard of management, yet complete removal is not always feasible due to tumor location, patient comorbidities, or the risk of surgical complications. In such contexts, radiation therapy (RT) has become an indispensable therapeutic tool, serving as a primary modality in select cases or as an adjunct for post-operative residual or recurrent disease.

Over the past two decades, radiation therapy for benign intracranial tumors has undergone a profound transformation, shaped by advances in imaging, treatment planning, and delivery technologies. This Research Topic brings together five important contributions that illustrate the evolving strategies, persistent challenges, and future prospects in this field, ranging from innovative dosimetric approaches and risk-mitigation strategies to case reports that underscore clinical complexity and multidisciplinary collaboration. Collectively, these studies highlight both the progress achieved and the work that remains to be done in balancing tumor control with preservation of normal tissue function and long-term quality of life.

Xiong et al. provide a key evaluation of HyperArc (HA), a novel automated non-coplanar volumetric modulated arc therapy (VMAT) technique, in the treatment of optic nerve sheath meningiomas. These tumors exemplify the challenge of managing benign lesions where the therapeutic window is narrow and their encasement of the optic nerve often precludes complete surgical resection without risking vision deficits. The study demonstrated that HyperArc offered superior target coverage and dose homogeneity compared with traditional coplanar VMAT techniques, while simultaneously achieving greater sparing of organs at risk (OARs), including the contralateral optic nerve, hippocampi, and lenses. These results underscore the value of automated, non-coplanar planning for tumors with tight therapeutic margins and highlight the potential to enhance reproducibility, efficiency, and dosimetric quality in clinical practice.

The importance of long-term safety is emphasized by Chao and Lee, who reviewed the risk of radiation-induced secondary malignancies in patients treated with the CyberKnife M6 radiosurgery system. By comparing multileaf collimator (MLC) and IRIS collimator plans, they found that MLC-based treatments achieved superior conformity and steeper dose gradients, thereby reducing estimated secondary cancer risks. Minimizing secondary cancer risk, especially in younger patients, is a critical consideration that should guide both clinical decision-making and future technological development. Their analysis underscores the importance of careful technology selection and meticulous planning when treating benign tumors in patients expected to have long survival.

Ungar et al. broaden the scope by providing a comprehensive review of emerging strategies for meningioma management, encompassing external beam radiotherapy, proton therapy, and brachytherapy, as well as the integration of artificial intelligence into treatment planning and delivery. Their synthesis highlights the advantages of advanced modalities such as proton therapy in reducing unnecessary dose to normal brain tissue and of brachytherapy in delivering highly focal treatment in selected cases. Particularly notable is their discussion of artificial intelligence, which promises to enhance contouring accuracy, streamline plan optimization, and potentially predict treatment outcomes or toxicity through radiomic and molecular data integration. The trajectory outlined in this review reflects the expanding vision of RT as both a technical and data-driven discipline, where precision is achieved not only through delivery but also through patient-specific planning.

Shifting from technological advancement to clinical complexity, the case reported by Muñoz et al. illustrates the diagnostic and management challenges in neuro-oncology. The authors describe a rare intracranial immature teratoma with secondary somatic malignant transformation into a Wilms tumor. Such a presentation underscores the diagnostic difficulties that can arise when tumors mimic benign pathology yet harbor malignant potential. The case illustrates the necessity of comprehensive histopathological assessment, vigilant clinical follow-up, and flexibility in individualized management. Although radiotherapy was not ultimately administered in this specific case, this report reinforces the indispensable role of radiation oncologists within multidisciplinary teams, who must remain prepared to adapt therapeutic strategies for rare and unpredictable clinical scenarios.

Zhang et al. report the successful management of a ruptured supratentorial dermoid cyst using a combined microscopic and neuroendoscopic approach, emphasizing the value of surgical innovation in reducing blind spots in the surgeon's field of vision and improving resection. While their case was managed purely with surgery, their report also reviews the role of radiotherapy as an adjuvant option for dermoid cysts, especially in cases of recurrence, malignant transformation, or incomplete removal. The authors note that radiotherapy can suppress the proliferative activity of cyst-lining cells to reduce content accumulation and the risk of recurrence. This highlights that, for patients with residual disease or those who are poor candidates for further surgery, adjuvant radiotherapy remains a valuable component of a multidisciplinary strategy.

Together, these contributions reflect the rapidly evolving landscape of radiation therapy for benign intracranial tumors. The integration of automation, non-coplanar planning, advanced collimation systems, and particle therapy have already enhanced therapeutic precision while reducing collateral toxicity, and the incorporation of artificial intelligence and molecular imaging into clinical workflows holds promise for truly individualized care. Despite these advances, significant challenges remain. Optimizing dose delivery while minimizing long-term sequelae is particularly demanding for tumors situated near OARs, and the accessibility and cost of advanced modalities such as proton therapy continue to limit their widespread adoption. Furthermore, prospective studies with long-term follow-up are essential to better characterize outcomes such as neurocognitive preservation, vascular health, endocrine function, and secondary cancer risk. Addressing these gaps will require sustained innovation, rigorous clinical evaluation, and continued multidisciplinary collaboration. As these tumors are often indolent but clinically impactful, patient-centered outcomes and quality of life must remain the ultimate benchmarks of success. This Research Topic highlights both the progress already achieved and the challenges that remain, underscoring the importance of ongoing dialogue across disciplines. With a collective commitment to precision, safety, and collaboration, the next era of radiation therapy will further refine treatment, improve survivorship, and enhance the quality of life for patients facing these deceptively benign yet clinically consequential tumors.

